# Global effects of progress towards SDGs on steeply sloped land use and land cover

**DOI:** 10.1016/j.fmre.2025.11.013

**Published:** 2025-11-24

**Authors:** Hongwei Zhang, Ji Chai, Zhihui Li, Haiyang Li, John Gibson, Pankaj Kumar, Gui Jin

**Affiliations:** a China University of Geosciences, Wuhan 430074, China; b Institute of Geographic Sciences and Natural Resources Research, Chinese Academy of Sciences, Beijing 100101, China; c School of Public Policy and Management, Tsinghua University, Beijing 100084, China; d Department of Economics, University of Waikato, Hamilton 3240, New Zealand; e Department of Geography, Delhi School of Economics, University of Delhi, Delhi 110007, India

**Keywords:** Steeply sloped land, Sustainable development goals, Effect, Land use/land cover change, PLS-SEM

## Abstract

Flat, highly suitable land for construction and cultivation is limited, and socio-economic development and population growth will surely lead to the exploitation of steeply sloped land, which is very difficult to use and extremely ecologically sensitive. Understanding how the trade-offs and synergies between economic development, social progress and ecological protection within the framework of the Sustainable Development Goals (SDGs) affect the exploitation and protection of steeply sloped land in an integrated manner should be an important part of land resources management, but is rarely addressed in existing research. Therefore, we produced global map of steeply sloped land use and land cover in 2020 with the spatial resolution of 10m×10m, and its distribution and structural characteristics were analyzed, the levels of achievement of the 17 sub-SDGs and these effects on steeply sloped land use and land cover structure were quantified. Results showed that: The distribution of steeply sloped land use and land cover around the world is extremely uneven. Area of steeply sloped land use and land cover in 54 countries exceeds 50%, and these countries or regions are mainly located in Asia and Europe. Global steeply sloped land use and land cover includes all types of commonly used land use and land cover classifications, which is mainly composed by tree cover, grassland, and bare/sparse vegetation, accounting for 48.663%, 21.418%, and 14.876% respectively, and cropland is the main land use type. the level of achieving the SDGs of essential needs and objectives shows the most significant negative direct effects on the amount of ecological space in the global steeply sloped land use and land cover system (−0.526, −0.318), the level of achieving the SDGs of governance has clear positive direct effects on the amount of construction space and ecological space (0.403, 0.324). The 17 sub-SDGs and the global steeply sloped land use and land cover structure also exert indirect effects through their interactions. Our conclusion is that it is necessary to strengthen the systematic monitoring of global steeply sloped land use and land cover, as well as the need for effective control of global steeply sloped land exploitation.

## Graphical abstract

**Figure:**
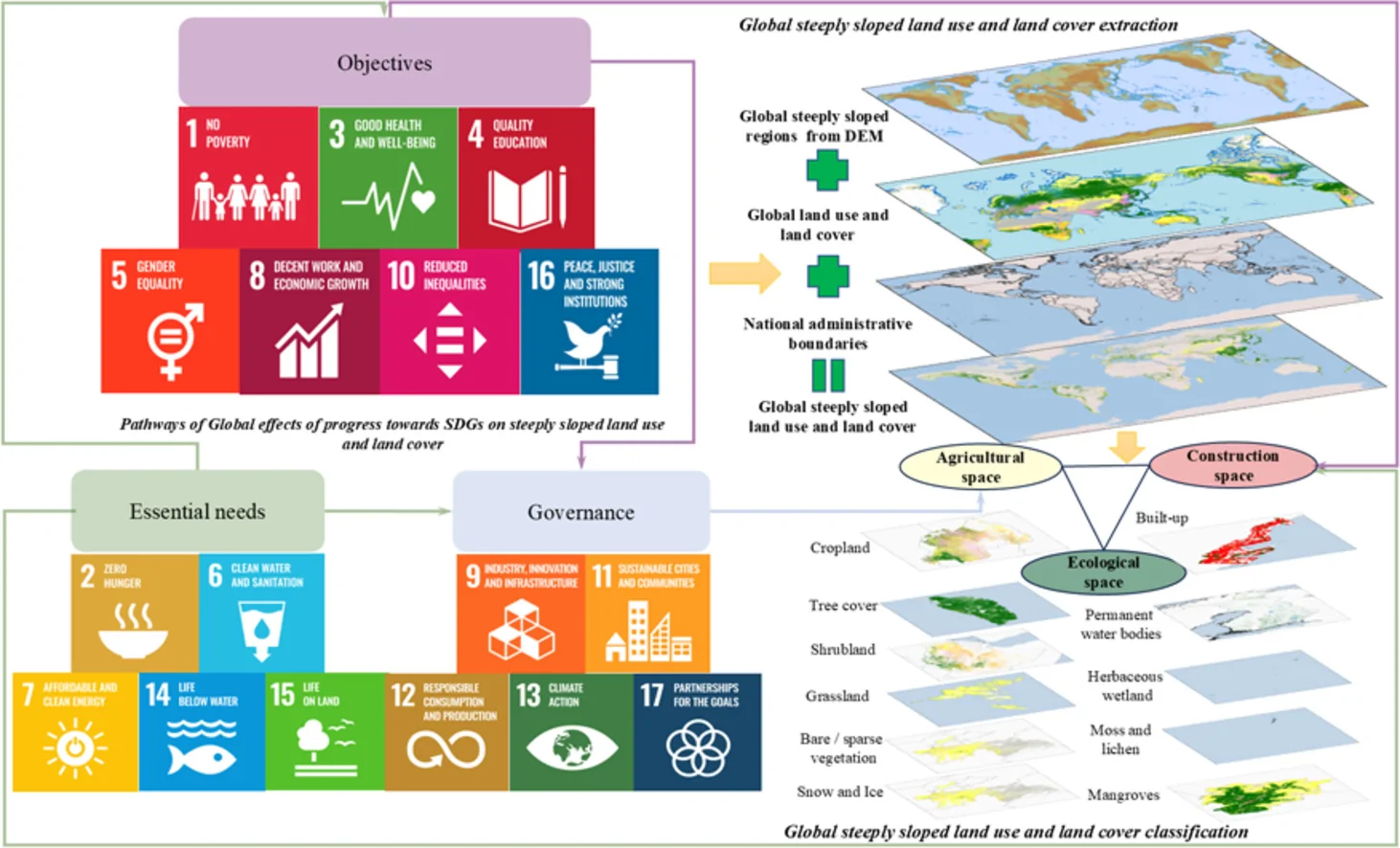

## 1 Introduction

Steeply sloped land refers to land surface with large slopes, consisting of different land use and land cover types, which are widely distributed in different countries or regions with extremely fragile ecological environments or in priority areas for ecological protection, where soil erosion, sharp reduction of biodiversity, and natural disasters caused by their exploitation can be more serious and more immediate [1,2]. The aim of sustainable development goals (SDGs) is to systematically address the fundamental relationships among society, ecology, economy, and the use of natural resources and to ensure that the most pressing development problems facing all humankind in the three dimensions of society, economy, and ecology are resolved in an integrated manner [[3], [4], [5]]. SDGs are closely linked to land use and land cover change and rational land use planning and management are the key to achieving economically, socially, and ecologically sustainable development [[6], [7], [8]]. In terms of economy, optimizing land use and land cover can promote industrial development and eliminate poverty [9,10]; on the social side, rational land use and land cover can improve the quality of life of residents and promote healthy lifestyles [11,12]; on the ecological side, scientific control measures about land use and land cover can protect the ecological environment and cope with climate change [13,14]. Meanwhile, as SDGs continue to be advanced globally, most of the SDGs that satisfy human survival, such as poverty eradication, hunger, and economic growth, may be given higher priority [[15], [16], [17], [18]], and superimposed on the advancement of engineering technology in human societies and the limited availability of land that is more suitable for agricultural development and construction, the exploitation of steeply sloped land in the SDGs process may be more serious than we have thought. However, little attention has been paid to this issue in existing studies.

In order to promote sustainable development on a global scale, the United Nations has broken down this ambitious SDGs into 17 sub-goals, each of which addresses a specific social, economic or environmental issue and aims to achieve comprehensive, inclusive, and sustainable development [19]. In order to ensure that the effectiveness of the implementation of these SDGs can be accurately assessed, a large number of scholars or institutions have also constructed an extensive system of monitoring indicators to track and report on the progress of countries or regions in SDGs by regularly collecting and analyzing data [3,20]. In the process of advancing these SDGs, researchers have gradually discovered an important phenomenon: different sub-goals do not exist in isolation from each other, and there is a complex interaction among them [21,22]. On the one hand, the realization of some sub-goals may be constrained by the implementation of other sub-goals, the so-called "trade-off" [15,23], on the other hand, there is also a tendency for some sub-goals to promote each other [[24], [25], [26]]; the existence of such trade-offs and synergies makes the achievement of the SDGs more complex and challenging [27,28]. In order to better understand and address these complex interactions, the researchers categorized 17 sub-goals into three categories: essential needs (SDGs 2, 6, 7, 14, and 15), objectives (SDGs 1, 3–5, 8, 10, and 16), and governance (SDGs 9, 11–13, and 17), which helps to get a clearer picture of the intrinsic linkages and prioritization among the sub-goals [19,29]. While the full realization of all the sub-goals still faces many difficulties and challenges until 2030, it is worth acknowledging that significant progress has been made on many of the SDGs over time. Such progress is not only reflected in specific data indicators, but has also profoundly changed people's lifestyles and values and raised the level of human well-being globally [30,31].

Studies of land use and land cover change have pointed out that economic and social development are the central drivers of land use and land cover change in different countries or regions, a process that is accelerating, inspired by the global ambition of SDGs [32,33]. The wave of global urbanization and explosive population growth has been marked by a dramatic expansion of construction land, often at the expense of highly utilized agricultural land in the first place [34]. The continued demand of agricultural land has forced humans to turn to pristine, ecologically vital, and fragile ecological space, and this process is manifested in the exploitation of steeply sloped land [[35], [36], [37]]. It is not only a visual manifestation of land use and land cover change but also has far-reaching impacts on local climates and exacerbates environmental problems such as soil erosion, highlighting the delicate and complex challenge of balancing development and conservation [[38], [39], [40]]. In summary, studying the effects of sustainable development goals on the structure of steeply sloped land use and land cover is of great practical significance.

Overall, economic and social development inspired by the SDGs has made remarkable progress, and a basic consensus has been reached on the widespread exploitation of steeply sloped land as a result of land use climbing driven by human economic and social development. However, related studies have focused on single dimensions such as steeply sloped cropland and construction land climbing, and there has been no research on how the SDGs can be continuously trade-off and synergized to integrate the exploitation of steeply sloped land, which in turn cannot effectively guide human beings to effectively control or scientifically exploit steeply sloped land. Therefore, the research objectives of this paper are: Firstly, to extract global steeply sloped land and analyze its spatial distribution. Secondly, to analyze the structure of steeply sloped land use and land cover and thus quantify the current status of steeply sloped land use and land cover. Thirdly, to determine the comprehensive effects of the 17 sub-goals of SDGs on the structure of steeply sloped land use and land cover. Fourthly, to clarify the direct and indirect effects of the achievement levels of the different categories of SDGs on the distributions of steeply sloped land use and land cover.

## 2 Methods

### 2.1 Digital Elevation Model

The highest spatial resolution of Digital Elevation Model (DEM) available and shared on a global scale is 30m×30m, including AW3D30 DSM, ASTER GDEM, and SRTM1 DEM, and scholars from countries in Europe, South America, Asia, and Africa have verified the accuracy of different DEM datasets. Results show that AW3D30 DSM has higher overall accuracy than ASTER GDEM and SRTM1 DEM, and AW3D30 DSM has advantages in expressing terrain details [[41], [42], [43]], which is more suitable for global-scale topography research or related data extraction and is transferred from the website of https://www.eorc.jaxa.jp/ALOS/en/dataset/aw3d30/aw3d30_e.htm. In this study, AW3D30 DSM would be used as the data source for the extraction of steeply sloped land use and land cover.

### 2.2 Global steeply sloped land use and land cover map

Global land use and land cover maps are another key variable for extracting global steeply sloped land use and land cover. The world's mainstream land use and land cover datasets covering 2020 include FROM_GLC, GLC_FCS30-2020, Globeland30, Esri Land Cover 10 m, and ESA World Cover 10 m 2020 V100. Accuracy research show that the ESA World Cover 10 m 2020 V100 dataset has higher accuracy on global scale (https://worldcover2020.esa.int/) [[44], [45], [46], [47]]. Therefore, according to the standard that relevant scholars consider cropland with a slope greater than 10° to be steeply sloped cropland [1], the research used the GEE platform to superimpose the extraction regions of slopes greater than 10° from AW3D30 DSM and ESA World Cover 10 m 2020 V100 to form global steeply sloped land use and land cover map. At the same time, since the spatial resolution of both the DEM dataset and global land use and land cover dataset used are very reliable, the accuracy of global steeply sloped land use and land cover maps does not need further verification. On the other hand, although the high resolution of steeply sloped land use and land cover that this paper extracted and mapped covers all countries or regions around the world, considering that this paper explored their relationships with the level of the attainment of 17 sub-goals related to steeply sloped land use and land cover, and the more complete monitoring of the attainment of SDGs only covers 164 countries or regions, except for in the parts of the distribution of global steeply sloped land and the structure of global steeply sloped land use and land cover, other parts of this study analyzed 164 national or regional samples. Compared with the area of global steeply sloped land use and land cover, this paper pays more attention to the proportion of steeply sloped land use and land cover to the total area of land use and land cover in different countries or regions. This proportion determines the endowment of land resources that is extremely difficult to use, and the formula is as follows.

(1) $$P_{a}=\frac{\sum_{b=10}^{n}S_{ab}}{L_{a}}$$

$P_{a}$ represents the proportion of steeply sloped land use and land cover to the total area of land use and land cover, $S_{ab}$ represents the area of *b*-type steeply sloped land use and land cover in *a*-th country or region. $L_{a}$ represents the total area of land use and land cover in the *a*-th country or region.

### 2.3 Estimating the structure of global steeply sloped land use and land cover

The structure of steeply sloped land use and land cover can represent the degree of human utilization of steeply sloped land and the possibility of development. Therefore, this paper calculated the proportion of different types of steeply sloped land use and land cover in the overall steeply sloped land use and land cover of each country or region, and the formula is as follows.

(2) $$S_{i}=\frac{S_{ab}}{\sum_{b=10}^{n}S_{ab}}$$

On the basis of calculating the proportion of different types of steeply sloped land use and land cover in the overall steeply sloped land use and land cover of each country or region, combined with land use and land cover classification of FAO and research results of land use and land cover type classification, based on whether there may be a closer potential relationship with human activities, the types of steeply sloped land use and land cover were classified into agricultural space, construction space and ecological space [46,48,49], where agricultural space incudes the type of cropland, construction space incudes the type of built-up, and ecological space includes the types of shrubland, grassland, bare/sparse vegetation, tree cover, snow and ice, permanent water bodies, Herbaceous wetland, mangroves, and moss and lichen, and indexes S*_agricultural space_*, S*_construction space_* and S*_ecological space_* are set.

### 2.4 Evaluative indexes for the level of the attainment of SDGs

Since the SDGs were proposed, scholars have conducted a series of studies on evaluative indexes for the level of the attainment of SDGs, and many indexes have been incorporated into the monitoring systems of Food and Agriculture Organization of the World, World Bank, World Health Organization, and Sustainable Development Report (https://dashboards.sdgindex.org/). Based on the comprehensive analysis of 17 sub-goals, a composite score of the 17 sub-goals was chosen to characterize the level of the attainment of SDGs [20]. On the other hand, from the perspective of maximizing benefits with minimum inputs through appropriate governance measures, studies have shown that the 17 sub-goals can be categorized into three categories: essential needs (SDGs 2, 6, 7, 14, and 15), objectives (SDGs 1, 3–5, 8, 10, and 16), and governance (SDGs 9, 11–13, and 17), which has been widely used in various SDGs studies [50,51], and this study would quantify the extent to which the SDGs have been achieved on the basis of the 17 sub-goal scores using the above categorization.

### 2.5 The PLS-SEM model specification

The PLS-SEM model is reduced form of the Partial Least Square (PLS)-Structural Equation Model (SEM). PLS seeks the best match between the data and the function by minimizing the variance, while SEM is a multivariate statistical method including regression analysis, factor analysis, path analysis, and multivariate analysis of variance (ANOVA), and is capable of hypothesis testing of theories. The advantages of PLS-SEM model are that (1) it requires only a minimum of 30 samples, (2) it does not require the input data to obey normal distribution, and (3) it is simultaneously applicable to reflective, formative, or a mixture of both measurable models [52,53]. Similar to principal component analysis, PLS-SEM model also has the ability to convert a large number of indexes into several composite indexes for dimensionality reduction, the measurable variables are close to the original indexes in terms of significance, and the latent variables are close to the principal components in terms of significance, and the modelling process needs to carry out factor analysis, constructive reliability and validity analysis, as well as discriminant validity analysis test, which can solve the problem of information overlap between indexes.

This paper took 164 countries or regions around the world with complete data on the level of the attainment of the 17 sub-goals as samples and constructed a PLS-SEM model with three categories of the 17 sub-goals: essential needs, objectives, and governance as the latent independent variables, and the percent of construction space, agricultural space, and ecological space in the steeply sloped land use and land cover as the latent dependent variables, in accordance with the results of the theoretical analysis of potential effects of progress towards SDGs on the structure of steeply sloped land use and land cover, in which all latent variables are reflective (Fig. 1). According to the quality control criteria for reflective models, Cronbach's Alpha (CA) and Composite Reliability (CR) were chosen to measure the internal consistency reliability of all latent variables, Average Variance Extracted (AVE) was chosen to measure the convergent validity of the measured variables within all latent variables, and the R-Squared for each latent variable measured the explanatory power of the internal measures of the latent variables. Calculations show that all latent variables of the structural model passed internal consistency reliability and convergent validity, and the measures had high explanatory power for all latent variables, as assessed in the Supplementary Material.

**Fig. 1:**
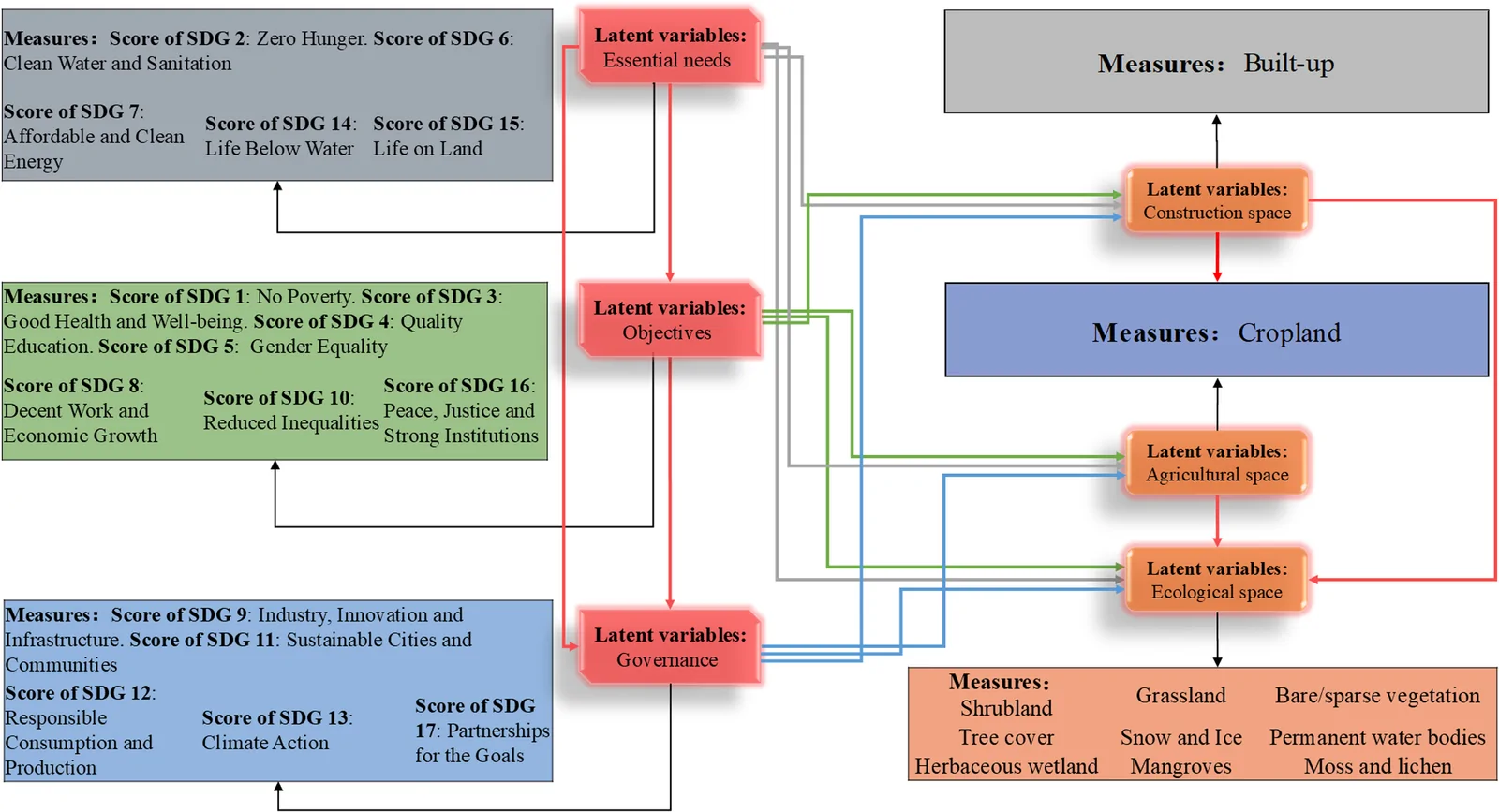
**Setting of latent variables and measures and the pathways of theoretical action**.

## 3 Results

### 3.1 Distribution of global steeply sloped land use and land cover

Spatial resolution and land use and land cover classification of global steeply sloped land use and land cover map in 2020 is consistent with the ESA World Cover 10 m 2020 V100 dataset, which is 10m×10m (Fig. 2a). Statistics on the area of steeply sloped land use and land cover in different countries or regions revealed that countries with larger areas, such as China, Canada, United States, Russia, and Brazil, also had larger areas of steeply sloped land use and land cover in 2020. At the same time, Asia is the main continent where countries or regions with larger areas of steeply sloped land use and land cover are distributed, and there are 31 countries or regions in the top 100, followed by Africa with 26 countries (Fig. 2b). Results of the proportion of steeply sloped land use and land cover in different countries or regions show that the proportion of steeply sloped land use and land cover in most countries or regions is <50%. However, the area of steeply sloped land use and land cover in 54 countries or regions exceeds 50% in the samples of 184 countries or regions around the world, and these countries or regions are mainly located in Asia and Europe (Fig. 2c).

**Fig. 2:**
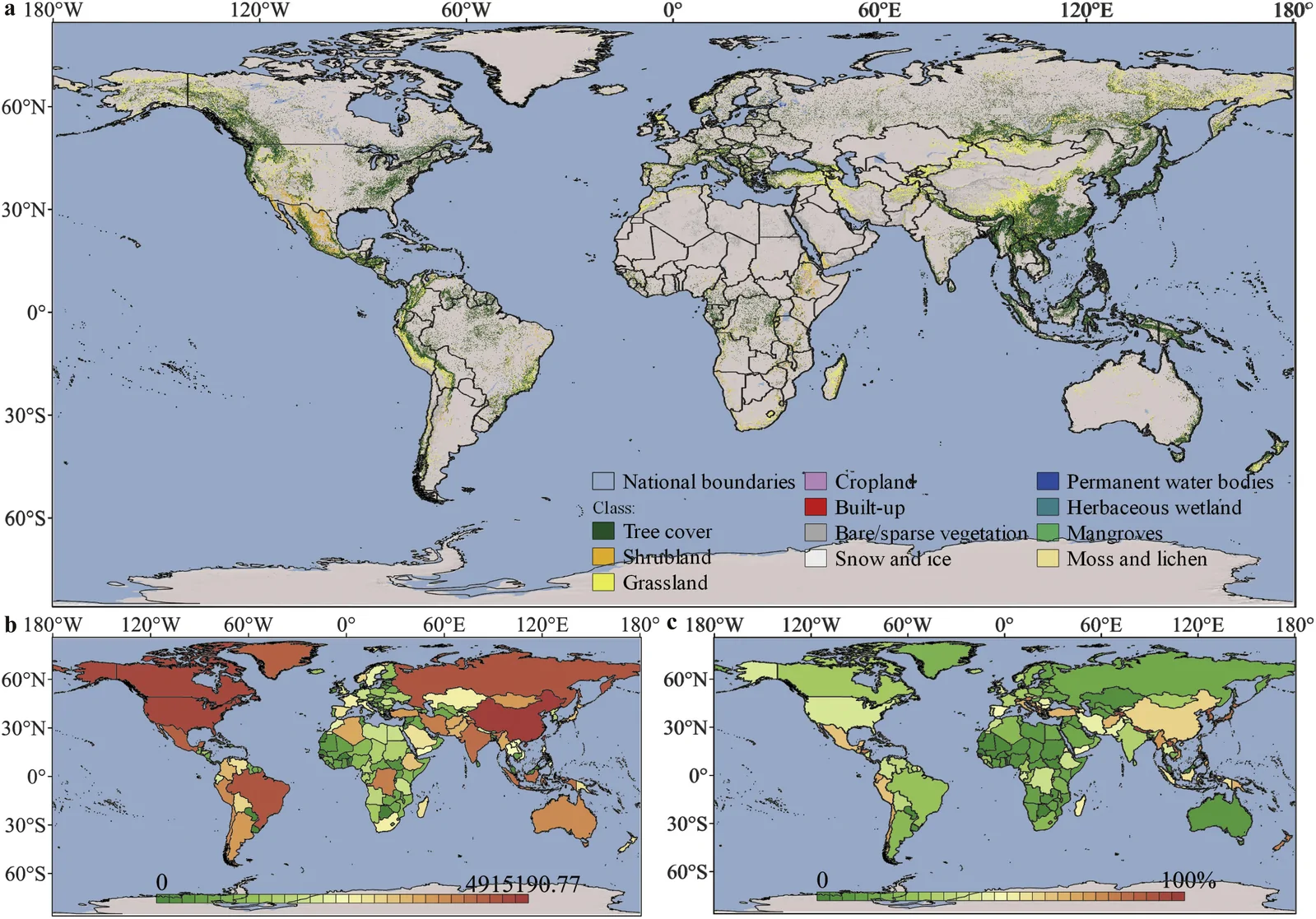
**Distribution of global steeply sloped land use and land cover, area of steeply sloped land use and land cover, and the proportion of steeply sloped land use and land cover to the total area of land use and land cover of 2020 in different countries or regions (*N* = 184).** (a) Extraction results of global steeply sloped land use and land cover. (b) Spatial distribution of the area of steeply sloped land use and land cover in different countries or regions (Index: S*area*). (c) Differences in the proportion of steeply sloped land use and land cover to the total area of land use and land cover area in different countries or regions (Index: S*percent*). (Map Review No.: GS(2021)648)

### 3.2 Structure of global steeply sloped land use and land cover

The structure of steeply sloped land use and land cover can reflect the surface coverage of steeply sloped land and human utilization of steeply sloped land. Global steeply sloped land use and land cover is mainly composed of tree cover, grassland, and bare/sparse vegetation, accounting for 48.663%, 21.418%, and 14.876%, respectively. The area of cropland also reached 1.318% (Fig. 3a). However, there are large differences in the proportion of steeply sloped land use and land cover types of shrubland, grassland, cropland, and bare/sparse vegetation in each country or region. Among the 184 countries or regions, there are 39 countries or regions with grassland accounting for more than 30% of the proportion of steeply sloped land use and land cover to the total area of land use and land cover, 16 countries or regions with shrubland accounting for more than 30%, 31 countries or regions with more than 30% in bare/sparse vegetation, and 52 countries or regions where the proportion of cropland in steeply sloped land use and land cover is less than 0.1% (Fig. 3b). `There are only 29 countries or regions whose built-up accounts for more than 1% (Fig. 3c). It should be noted that there are more countries or regions with a higher proportion of tree cover in steeply sloped land use and land cover, and 127 countries or regions have more than 30% of tree cover in steeply sloped land use and land cover. Snow and ice, permanent water bodies, herbaceous wetland, mangroves, and moss and lichen accounted for a small proportion in each country or region (Fig. 4d). On the other hand, the proportion of agricultural space, construction space, and ecological space is calculated; there are 10 countries or regions with agricultural space accounting for more than 10%, only 6 countries or regions with construction space accounting for more than 10%, and 183 countries or regions with ecological space accounting for more than 30%.

**Fig. 3:**
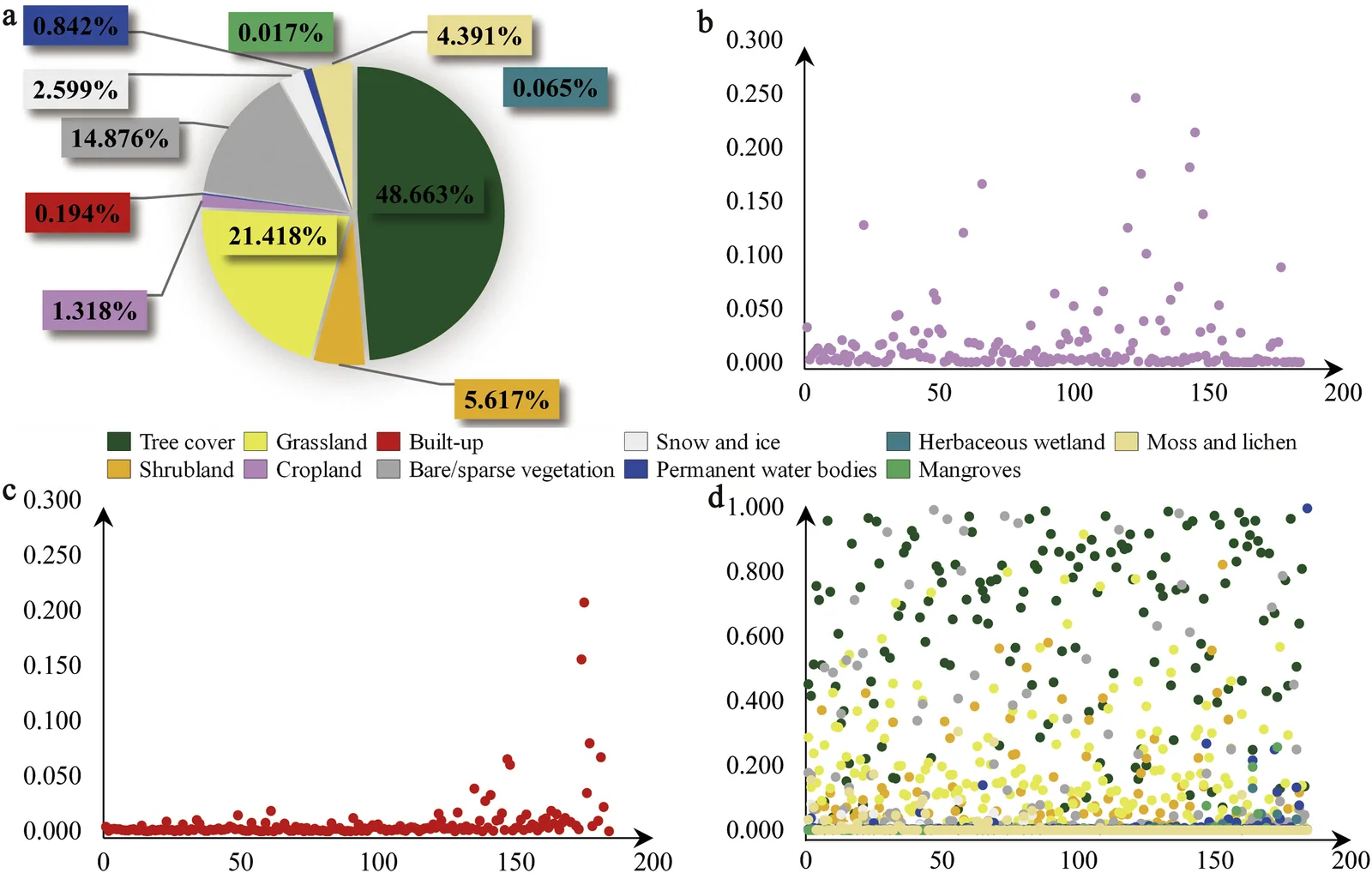
**Structural characteristics of global steeply sloped land use and land cover of 2020 in different countries or regions (*N* = 184).** X-axis: each country or region, Y-axis: the proportion of different steeply sloped land use and land cover types. (a) Composition and proportion of global steeply sloped land use and land cover. (b) Proportions of different steeply sloped land use and land cover types that constitute agricultural space in different countries or regions. Agricultural space (Index: S*agricultural space*) includes cropland. (c) Proportions of different steeply sloped land use and land cover types that constitute construction space in different countries or regions. Construction space (Index: S*construction space*) includes built-up. (d) Proportions of different steeply sloped land use and land cover types that constitute ecological space in different countries or regions. Ecological space (Index: S*ecological space*) includes shrubland, grassland, bare/sparse vegetation, tree cover, snow and ice, permanent water bodies, herbaceous wetland, mangroves, and moss and lichen.

**Fig. 4:**
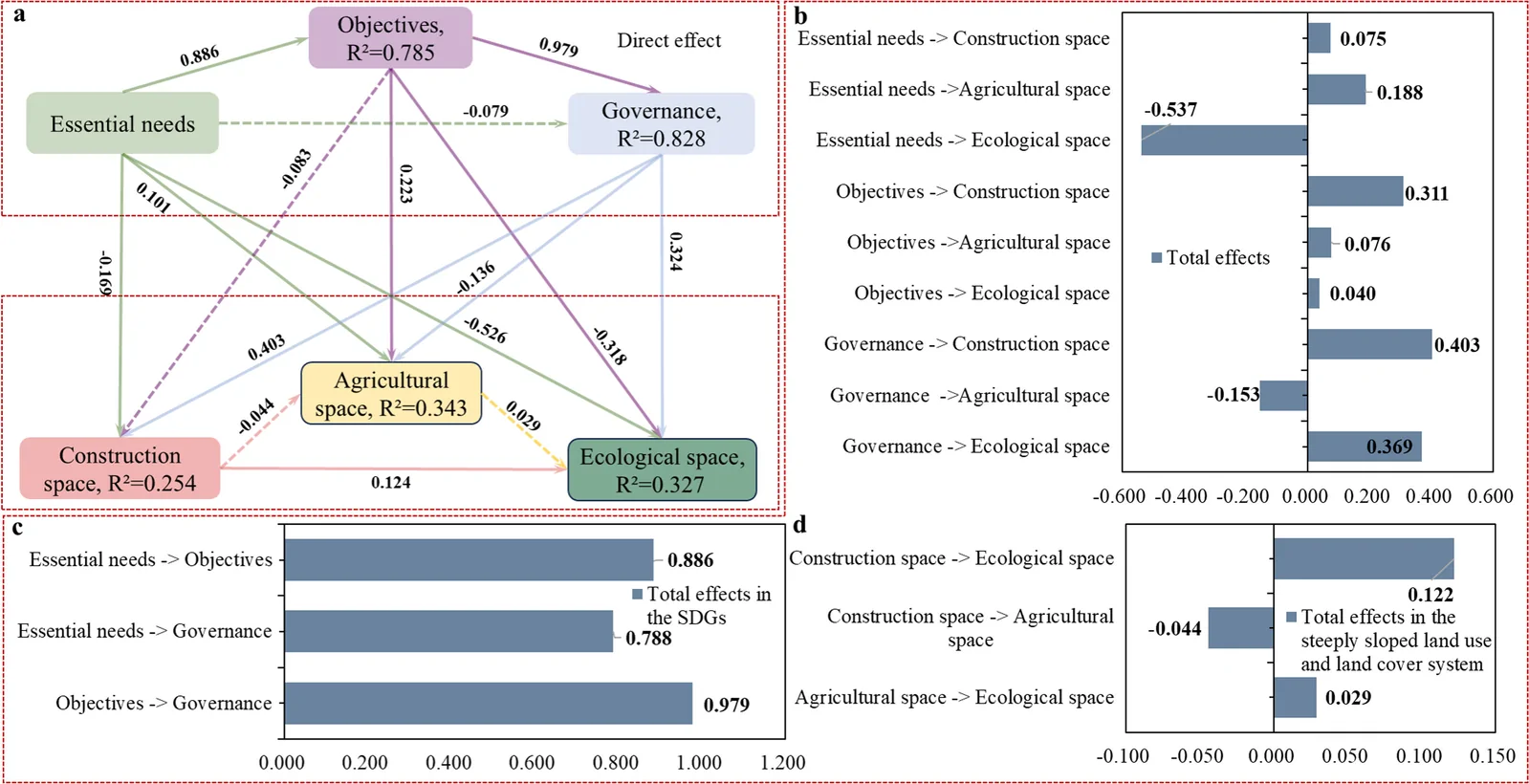
**PLS-SEM model showing the total effects of progress towards SDGs on global steeply sloped land use and land cover (*N* = 164).** (a) Direct path diagrams of three categories of SDGs on three types of space in global steeply sloped land use and land cover system; solid lines indicate strong direct effects, and dashed lines indicate weak direct effects. R² represents the explanatory power of measures in the outer model for the latent variables. (b) Standardized path coefficients of total effects from three categories of SDGs on three types of space. (c) Standardized path coefficients of total effects within three categories of SDGs. (d) Standardized path coefficients of total effects within three types of space.

### 3.3 Effects of progress towards SDGs on steeply sloped land use and land cover

Based on the theoretical analysis of the effects of progress towards SDGs on the structure of steeply sloped land use and land cover system: There are interactions among SDGs, and there are also interactions among construction space, agricultural space, and ecological space in the steeply sloped land use and land cover system comprising different land use and land cover types; and the structure of steeply sloped land use and land cover is affected by the combination of the interactions among SDGs. To effectively test the theoretical hypotheses, the PLS-SEM model was established, and the total effect, direct effect, and indirect effect of the three categories of essential needs, objectives, and governance in the SDGs and the role of construction space, agricultural space, and ecological space in the global steeply sloped land use and land cover system were measured.

Firstly, there are direct effect paths between essential needs, objectives, and governance in the SDGs, and the effect paths are positive. Secondly, the amount of construction space and the amount of ecological space in the global steeply sloped land use and land cover system show direct correlation, and the effect is positive. However, construction space and agricultural space, as well as agricultural space and ecological space, show little correlation. Thirdly, the level of achieving the SDGs of essential needs shows direct effects on the amount of agricultural space, construction space, and ecological space in the global steeply sloped land use and land cover system, but a positive direct effect on the amount of agricultural space and negative direct effects on the amount of construction space and ecological space; the level of achieving the SDGs of objectives has positive direct effect on the amount of agricultural space, but negative direct effects on the amount of ecological space; the level of achieving the SDGs of governance has clear positive direct effects on the amount of construction space and ecological space, but has clear negative direct effect on the amount of agricultural space (Fig. 4a).

Essential needs had negative total effects on ecological space, but positive total effects on agricultural space in the steeply sloped land use and land cover system; objectives had positive total effects mainly on construction space in the steeply sloped land use and land cover system; and governance had significant positive total effects on both construction and ecological space, but negative total effects on agricultural space in the steeply sloped land use and land cover system (Fig. 4b). Total effects include direct effects and indirect effects; there are positive total effects among three categories of essential needs, objectives, and governance in the SDGs. However, compared with the direct effects, the total effect that essential needs have on governance is much larger and is positive (Fig. 4c). The main functional relationship among construction space, agricultural space, and ecological space in the steeply sloped land use and land cover system is the positive relationship between construction land and ecological space (Fig. 4d).

The sub-category of essential needs in the SDGs has positive total indirect effects on construction space in the global steeply sloped land use and land cover system and the sub-category of governance. The sub-category of objectives in the SDGs has a positive total indirect effect on construction space and ecological space in the global steeply sloped land use and land cover system, but has a negative total indirect effect on agricultural space. In terms of specific indirect effects, there are positive specific indirect effects among essential needs, objectives, and governance, and the positive specific indirect effects of which can be extended to the amount of construction space and ecological space in global steeply sloped land use and land cover system, and negative specific indirect effects of which can be extended to the amount of agricultural space. There are also complete specific indirect effects between essential needs, objectives, and the amount of agricultural space (positive) and ecological space (negative) in steeply sloped land use and land cover system. At the same time, objectives and governance have specific indirect effects on construction space (positive), agricultural space (negative), and ecological space (positive) in global steeply sloped land use and land cover system (Table 1).

**Table 1:** **Standardized total indirect effect, and specific indirect effect sizes of progress towards SDGs on steeply sloped land use and land cover**.

Latent variable	Effect	Path coefficient
Essential needs ->Governance	Total indirect effect	0.867
Objectives ->Construction space	Total indirect effect	0.394
Objectives ->Ecological space	Total indirect effect	0.358
Essential needs ->Construction space	Total indirect effect	0.244
Objectives ->Agriculture space	Total indirect effect	−0.147
Essential needs ->Objectives ->Governance	Specific indirect effect	0.867
Objectives ->Governance ->Construction space	Specific indirect effect	0.394
Essential needs ->Objectives ->Governance ->Construction space	Specific indirect effect	0.349
Objectives ->Governance ->Ecological space	Specific indirect effect	0.317
Essential needs ->Objectives ->Governance ->Ecological space	Specific indirect effect	0.281
Essential needs ->Objectives ->Agriculture space	Specific indirect effect	0.197
Essential needs ->Objectives ->Governance ->Agriculture space	Specific indirect effect	−0.118
Objectives ->Governance ->Agriculture space	Specific indirect effect	−0.133
Essential needs ->Objectives ->Ecological space	Specific indirect effect	−0.282

## 4 Discussion

### 4.1 Global steeply sloped land use and land cover mapping

Global high spatial resolution steeply sloped land use and land cover maps in 2020 were produced. Although scholars had tried to extract steeply sloped cropland or steeply sloped agricultural land and to research urban construction land use climbing [2,54], the spatial resolution of the data involved in the global scale was low and just related to a single land use or land cover type [1]. However, this does not effectively characterize the distribution patterns of different land use and land cover types across the steeply sloped land use and land cover system when different land use and land cover distributions had been shown to be correlated. With the production of a series of high spatial resolution land use and land cover datasets, this study attempted to extract global steeply sloped land use and land cover covering all land use and land cover types, compensating for the fact that no scholars had systematically extracted spatial data on steeply sloped land use and land cover including all types of land uses and land cover. The resulting global steeply sloped land use and land cover data can provide important data production ideas for research on global steeply sloped agricultural development and the impact of steeply sloped land development on global soil erosion or terrestrial biodiversity [40,55]. It can provide the basis for identifying the extent of human activities and steeply sloped land exploitation in different countries or regions of the world.

Distribution of global steeply sloped land use and land cover is extremely uneven in different countries or regions, the larger area of land use type in steeply sloped land use and land cover that is closely related to human activities is cropland at the global scale. Although the larger the country, the more steeply sloped land there is, most of the larger countries have a smaller proportion of steeply sloped land use and land cover in terms of the proportion of steeply sloped land to the total area of land use and land cover. The composition of steeply sloped land use and land cover shows that all types of land use and land cover in the earth 's surface system are involved, and many agricultural productive activities have been extended to steeply sloped areas, which is manifested by the fact that cropland related to human survival is an important component of global steeply sloped land use and land cover, but tree cover is the main component of steeply sloped land use and land cover in most countries or regions. Considering that ecological space with tree cover as the core is the main component of steeply sloped land use and land cover system, the existence of agricultural space will inevitably bring about the reduction of ecological land at the global scale, so special attention needs to be paid to the proportion of agricultural space in global steeply sloped land use and land cover system.

### 4.2 Effect paths within different categories of SDGs and global steeply sloped land use and land cover system

There are multifarious effects within different categories of SDGs and global steeply sloped land use and land cover system at the national scale. The study validated the roles among three categories of SDGs, and there are very strong effect paths in the categories of essential needs, objectives, and governance, in which essential needs to objectives and objectives to governance show strongly positive direct effects, respectively, but essential needs to governance show significantly positive indirect effects. This indicates that there is a prioritization from surviving to thriving, which is consistent with previous studies that prioritization is required for the achievement of SDGs, such as the problem of hunger, which should be solved first [15,22,56]. On the other hand, positive direct effects existed only for the amount of construction space and ecological space in the steeply sloped land use and land cover system at the national scale, and although agricultural space occupies a large total area that related to human activities on a global scale, its statistical correlation with construction space and ecological space is not strong at the national scale, which differed from the combined distribution of construction space and agricultural space in the land use and land cover system from the generalized findings in land use and land cover change studies that construction space encroaches on both agricultural space and ecological space, agricultural land encroaches on ecological space, and that both agricultural land and construction land encroach on ecological space [57,58]. The deeper reason may be that humans can obtain sufficient ecological products from ecological space such as forests as food or in exchange for food. Policy makers need to consider the overall distribution of different types of steeply sloped land use and land cover and the measurement and mapping of their changes, as well as the interactions among SDGs across different categories, in order to comprehensively monitor potential constraints or risks that may arise from the exploitation of steeply sloped land.

### 4.3 Effects of progress towards SDGs on steeply sloped land use and land cover

Essential needs, objectives, and governance of SDGs show obvious effects on the distribution of construction space, agricultural space, and ecological space in steeply sloped land use and land cover system. The level of the attainment of governance on the amount of construction space in the global steeply sloped land use and land cover system is manifested as a positive direct effect. This shows that governance-related SDGs have promoted the rational planning and management of steeply sloped land use and land cover, and when formulating policies, the government and relevant agencies would consider the rational layout of construction space to avoid disorderly construction on steeply sloped regions. Objectives are manifested as a positive indirect effect, which shows that the ultimate realization of objectives-related SDGs requires an amount of construction space to support it. Essential needs are manifested as a weak negative direct effect, which suggests that there will be some limitations on the development of construction space in order to meet these essential needs-related SDGs in steeply sloped regions.

While the level of the attainment of governance on the amount of agricultural space in the global steeply sloped land use and land cover system is manifested as negative direct effect, the reason for this phenomenon is that governance emphasizes sustainability and science, and in steeply sloped regions, it will limit unreasonable agricultural reclamation practices. Objectives are manifested as negative indirect effect and positive direct effect, the process of realizing some of the goals will prompt people to move to non-agricultural industries and reduce their dependence on agriculture, which will indirectly lead to a lower demand for agricultural space from the perspective of negative indirect effects; it may be possible to rationalize the planning of suitable agricultural space in steeply sloped regions in order to provide healthy agricultural products and create agricultural-related employment opportunities, so it will in turn have a positive impact on the amount of agricultural space from the perspective of positive direct effects. Essential needs are manifested as positive direct effect, since the essential needs requires food security and so on, a certain amount of agricultural space is needed to grow food crops and so on, even in steeply sloped regions, agricultural space is being developed to meet essential needs-related SDGs through agricultural technology and soil and water conservation measures, thereby contributing to an increase in the amount of agricultural space.

The level of the attainment of governance on the amount of ecological space in the global steeply sloped land use and land cover system is manifested as positive direct effect. This is because governance-related SDGs all focus on the protection and restoration of the ecological environment, steeply sloped regions are ecologically important regions, and by strengthening ecological management, the total amount of ecological space is directly increased and the quality of the ecological environment is improved. Objectives are manifested as negative direct effect and positive indirect effect. In terms of negative direct effects, some objectives-related SDGs may encroach on ecological space in the pursuit of economic development. However, the positive indirect effect lies in the fact that the realization of objectives-related SDGs such as quality education (Goal 4) and gender equality (Goal 5) can enhance people's awareness of environmental protection and ecological literacy so that they will be more conscientious about protecting ecological space, which will be conducive to the maintenance and increase of the total amount of ecological space. Essential needs are manifested as negative direct effect. Although essential needs-related SDGs are for the protection of the ecological environment, some water conservancy facilities and so on may be constructed in steeply sloped regions in practice, causing some disturbance and damage to the original ecological space and leading to a negative change in the total amount of ecological space. We need to pay attention to the compounding effect of economic and social development goals on the exploitation of global steeply sloped land in the course of research on the effects, laws, and management of steeply sloped land exploitation.

## 5 Conclusion

By mapping the distribution of global steeply sloped land use and land cover, it is found that countries or regions with more steeply sloped land are mainly located in Asia, North America, and Europe, and these countries or regions with larger national land also have more steeply sloped land, but the proportion of steeply sloped land in the national land shows that many of the countries or regions that account for more steeply sloped land are those with smaller national land. In terms of continents, Asia has the largest proportion of steeply sloped land at 41.96%, North America has 28.99% of steeply sloped land, South America has 11.04%, Africa has 9.68%, Europe has 6.34%, and Oceania has the smallest proportion of steeply sloped land at 1.99% in 2020.

Although forest land, which is least affected by human activities, is the dominant land use and land cover type in steeply sloped land use and land cover system, land use that are closely related to human production and livelihoods are already widely distributed in steeply sloped regions, which has encroached heavily on ecological land represented by forest land. Since forest land are important for water retention and soil conservation, this situation will pose a great challenge to the management of ecological and environmental problems closely related to land use and land cover change on steeply sloped regions, such as soil erosion and landslides, in each country or region.

Achieving high levels of the SDGs in the essential needs category has a significant, total negative effects on the amount of ecological space in steeply sloped land use and land cover system. Achieving high levels of SDGs in the objectives category will have a significant, total positive effect on the amount of steeply sloped construction space. Achieving high levels of the SDGs in the governance category also shows significant, total positive effects on the amount of steeply sloped construction space. The above suggests that great ecological risks may result when solving the problem of subsistence. Under the premise of solving the problem of subsistence, human beings will not spend huge amounts of costs to exploit steeply sloped land for agricultural production activities, but in order to pursue better living conditions and ecological environments, human beings are willing to exploit steeply sloped land with high investment for construction and ecological protection at the same time.

The process by which the level of achievement of essential needs, objectives, and governance in the SDGs influences the structure of steeply sloped land use and land cover system is not always direct, there are also indirect effects. There are clear pathways between the essential needs, objectives, and governance in the SDGs, which are closely related to the amount of ecological space, agricultural space, and construction space in steeply sloped land use and land cover system. While effectively promoting synergies between global economic and social development and ecological and environmental protection using the SDGs as a consensus, it is important to consider their effects on steeply sloped land use and land cover.

## Data availability

All data used in the analysis are publicly accessible. Global land cover and land use change dataset is available at https://viewer.esa-worldcover.org/worldcover; DEM dataset is available at https://www.eorc.jaxa.jp/ALOS/en/dataset/aw3d30/aw3d30_e.htm. Global administrative boundary dataset is available at Amap Open Platform (https://lbs.amap.com/api/javascript-api-v2/guide/layers/districtlayer).

## CRediT authorship contribution statement

**Hongwei Zhang:** Writing – original draft, Visualization, Software, Methodology, Funding acquisition, Formal analysis, Data curation, Conceptualization. **Ji Chai:** Writing – original draft, Software, Funding acquisition, Formal analysis. **Zhihui Li:** Writing – review & editing, Writing – original draft, Validation, Supervision, Software, Funding acquisition, Conceptualization. **Haiyang Li:** Visualization, Software, Formal analysis, Data curation. **John Gibson:** Writing – review & editing, Visualization, Validation, Software, Conceptualization. **Pankaj Kumar:** Writing – review & editing, Validation, Supervision, Conceptualization. **Gui Jin:** Writing – review & editing, Validation, Software, Project administration, Methodology, Funding acquisition, Conceptualization.

## Declaration of competing interest

The authors declare that they have no conflicts of interest in this work.
